# Identification and Functional Prediction of Long Intergenic Non-coding RNAs Related to Subcutaneous Adipose Development in Pigs

**DOI:** 10.3389/fgene.2019.00160

**Published:** 2019-03-04

**Authors:** Gaoli Shi, Lin Chen, Guoting Chen, Cheng Zou, Jingxuan Li, Mengxun Li, Chengchi Fang, Changchun Li

**Affiliations:** ^1^Key Laboratory of Agricultural Animal Genetics, Breeding and Reproduction of the Ministry of Education and Key Laboratory of Swine Genetics and Breeding of the Ministry of Agriculture, Huazhong Agricultural University, Wuhan, China; ^2^The Cooperative Innovation Center for Sustainable Pig Production, Wuhan, China

**Keywords:** lincRNA, RNA-seq, subcutaneous fat development, backfat thickness, pig

## Abstract

An increasing number of studies have shown that long intergenic non-coding RNAs (lincRNAs) are a very important class of non-coding RNAs that plays a vital role in many biological processes. Adipose tissue is an important place for storing energy, but few studies on lincRNAs were related to pig subcutaneous fat development. Here, we used published RNA-seq data from subcutaneous adipose tissue of Italian Large White pigs and identified 252 putative lincRNAs, wherein 34 were unannotated. These lincRNAs had relatively shorter length, lower number of exons, and lower expression level compared with protein-coding transcripts. Gene ontology and pathway analysis indicated that the adjacent genes of lincRNAs were involved in lipid metabolism. In addition, differentially expressed lincRNAs (DELs) between low and high backfat thickness pigs were identified. Through the detection of quantitative trait locus (QTL), DELs were mainly located in QTLs related to adipose development. Based on the expression correlation of DEL genes and their differentially expressed potential target genes, we constructed a co-expression network and a potential pathway of DEL’s effect on lipid metabolism. Our study identified and analyzed lincRNAs in subcutaneous adipose tissue, and results suggested that lincRNAs may be involved in the regulation of subcutaneous fat development. Our findings provided new insights into the biological function of porcine lincRNAs.

## Introduction

Pigs are very similar to humans in terms of metabolic characteristics and organ development, so pigs are an ideal animal model for human disease research (Lunney, 2007; Schook et al., 2015). For example, atherosclerosis and diabetes are examples of the impact of pigs as biomedical model (Lunney, 2007). These diseases have become the focus of research because of the growing problem of obesity (Lunney, 2007). To delay disease progression and prevent disease occurrence, pigs are being extensively studied as animal models of these diseases to determine disease pathologies, such as the role of genetic background (Lunney, 2007). Meanwhile, pigs provide essential nutrients to humans, such as protein. With the improvement of human living standards, the demand for lean meat rate of pigs is also increasing. The 10th rib BFT and days to 100 kg are the economically important traits in pigs and are commonly used to predict carcass lean meat content and growth rate in pig breeding programs, respectively (Guo et al., 2017; Jiang et al., 2018). Furthermore, a previous study indicated that a low amount of subcutaneous fat or backfat deposition means good growth performance (Zambonelli et al., 2016).

LincRNAs are a class of non-coding RNAs (ncRNAs) that are transcribed between protein-coding gene regions and are more than 200 nucleotides (nt) in length (Han and Chen, 2013). In recent years, due to the development of sequencing technology, a large number of lincRNAs have been identified in many plants and animals (Guttman et al., 2010; Pauli et al., 2012; Lv et al., 2014; Hao et al., 2015; Zhou et al., 2015; Wang J. et al., 2016). Some lincRNAs had been identified in humans and mice, but most lincRNAs are still unknown (Chen X. et al., 2018). Moreover, studies have shown that lincRNAs play important roles in some biological processes, such as mouse embryonic development (Lv et al., 2013; Zhang et al., 2014), immune response (Carpenter et al., 2013a,b; Heward and Lindsay, 2014), human fat deposition (Liu et al., 2014; Ding et al., 2018; Gao et al., 2018), and human and pig muscle growth and development (Zhao et al., 2015; Gao et al., 2017; Lim et al., 2018).

In animals, adipose tissues play an important role and is the main energy storage site (Wang S. et al., 2016). Excessive accumulation of human adipose tissue can lead to obesity, further causing some chronic diseases, such as hypertension, diabetes, and cancer (Hanson et al., 2014; Rao et al., 2014). Studying the mechanism of adipose development cannot only improve the breeding efficiency of pigs but also help overcome obesity and related diseases. Previous studies found that some protein-coding genes, such as *CTRP6* (Wu et al., 2017), *NR1H3* (Zhang et al., 2016), *ubiquitin D* (Zhao et al., 2018), and *MAT2B* (Zhao et al., 2016), play an important role in regulating pig fat deposition. However, few studies are available on the mechanism of action of lincRNAs in pig fat deposition, and most functions of the lincRNAs in pig subcutaneous fat development are still unknown.

In this study, we used the published RNA-seq data from a previous study to assemble the transcriptome of subcutaneous adipose tissue in ILW pigs (Zambonelli et al., 2016). Zambonelli et al. (2016) measured the EBV in millimeter by using the BFT by the best linear unbiased prediction multiple-trait animal model program (Henderson and Quaas, 1976). The fixed effects of batch in test, gender, weight at slaughter, inbreeding coefficient, and the random effects of animal were all included in the model (Zambonelli et al., 2016). EBV was used for animal breeding selection and increased genetic gain in breeding programs (Kasinathan et al., 2015; Shin et al., 2017). ILW pigs were divided into two groups, namely, fat and lean samples, according to the difference of BFT EBV (Zambonelli et al., 2016). Based on the expression correlation of DEL genes and its neighboring protein-coding genes or DEPTGs, we investigated the role of lincRNAs in subcutaneous fat deposition of pigs. In summary, our study suggested that lincRNA plays an important role in pig subcutaneous fat development. This work enriches our knowledge on lincRNAs in pig and provides a valuable resource for future genetic and genomic studies.

## Materials and Methods

### Ethics Statement and the Datasets Source

All experiments in our study were performed according to the guidelines of the Key Lab of Agriculture Animal Genetics, Breeding, and Reproduction of Ministry of Education, Animal Care and Use Committee, Wuhan, China. In this study, the formula and amount of the ration were similar in animals used for RNA-seq (Zambonelli et al., 2016). Samples were taken from the subcutaneous adipose tissue of ILW pigs at an average age of 8 months (Zambonelli et al., 2016). 20 RNA-seq datasets were downloaded from the NCBI GEO databases with the accession numbers provided by Zambonelli et al. (2016) (Table 1, GEO accession GSE68007). All RNA-seq datasets were divided into two groups with 10 replicates in each group according to the difference of BFT EBV (Zambonelli et al., 2016).

**Table 1:** Summary of RNA-seq data.

Sample	Sex	BFT EBV value	Accession number	Raw reads	Mapping ratio (%)	Uniquely mapping ratio (%)
fat_1	Male	6.03	SRR1979630	180,176,718	94.88	74.56
fat_2	Female	7.17	SRR1979632	169,629,786	95.18	76.56
fat_3	Male	7.36	SRR1979633	192,010,306	94.79	72.25
fat_4	Female	5.05	SRR1979634	226,216,232	95.28	74.48
fat_5	Male	4.76	SRR1979635	192,017,336	94.85	71.49
fat_6	Female	5.75	SRR1979638	217,143,948	94.63	69.82
fat_7	Female	4.41	SRR1979639	186,887,716	94.77	72.89
fat_8	Male	3.27	SRR1979640	201,286,514	95.69	76.75
fat_9	Male	3.54	SRR1979645	217,308,832	94.69	70.77
fat_10	Female	4.88	SRR1979647	179,852,694	93.43	69.69
lean_1	Female	–7.54	SRR1979629	249,330,078	94.92	67.46
lean_2	Male	–8.03	SRR1979631	165,371,992	94.73	73.02
lean_3	Female	–10.59	SRR1979636	201,143,180	94.37	70.35
lean_4	Male	–9.91	SRR1979637	189,907,566	94.91	73.99
lean_5	Female	–7.82	SRR1979641	197,227,668	94.81	69.49
lean_6	Female	–10.27	SRR1979642	208,863,596	94.92	73.44
lean_7	Male	–7.61	SRR1979643	207,565,950	95.03	74.87
lean_8	Female	–10.37	SRR1979644	161,880,112	94.40	70.95
lean_9	Male	–6.46	SRR1979646	194,960,118	94.72	71.33
lean_10	Male	–7.71	SRR1979648	178,343,072	94.06	71.22

*BFT, backfat thickness; EBV, estimated breeding value.*

### Publicly Available Annotations

The pig gene annotations used in this article were downloaded from the Ensembl database at http://ftp.ensemblorg.ebi.ac.uk/pub/release-93/gtf/sus_scrofa/. Referring to previous studies, and combining ALDB^1^ with NONCODE database^2^, we obtained pig lincRNA annotations (Li et al., 2015; Tang et al., 2017; Zou et al., 2017a,b). In addition, the non-redundant reference sequence database was downloaded from https://ftp.ncbi.nih.gov/blast/db/.

### RNA-Seq Reads Mapping and Transcriptome Assembly

All RNA-seq reads were mapped to the pig reference genome (*Sus scrofa* 11.1^3^) using the default parameters of the HISAT2 version 2.0.1 (Pertea et al., 2016; Keel and Snelling, 2018; Kim et al., 2018). Then, we set the “-G” option of StringTie version 1.2.2 for transcript assembly, and obtained 20 samples of the GTF files respectively (Pertea et al., 2015, 2016). Afterward, the 20 GTF files were merged into a non-redundant transcriptome using the merge tool in the StringTie package (Pertea et al., 2016). The putative lincRNAs were then obtained by filtering the unique transcriptome from the lincRNA detection pipelines (Zou et al., 2017b).

### LincRNAs Identification Pipeline

Referring to our laboratory’s previous research methods (Zou et al., 2017b), the unique transcriptome assembled by the merging tool was gradually screened to obtain candidate lincRNAs. The method is as follows: (1) Retained those transcripts with ‘u’ category categorized by using gffcompare, which indicated intergenic transcripts. (2) The transcripts with length ≥ 200 bp and exon number ≥ 2 were retained. Because putative long non-coding RNAs (lncRNAs) were defined as transcripts that are more than 200 bp (Pauli et al., 2012; Ulitsky and Bartel, 2013). (3) Calculated the coding potential of transcripts by the CPC tool (Kong et al., 2007), retaining the transcripts that have a CPC score less than 0 which means that there was no coding potential. (4) Filtered out the transcripts which containing any known protein coding domain. Firstly, the sequences of transcript were translated into six possible protein sequences by using Transeq^4^. Then, the corresponding transcripts which had a significant hit in the Pfam database^5^ were discarded using HMMER (E-value < 1e-5) (Prakash et al., 2017). (5) BLASTX program (Mount, 2007) was used to filter out any transcripts that have similarities to known proteins in the NCBI NR and UniRef90 databases (E-value < 1e-5). (6) The FPKM values for the 20 samples were estimated using the “-B” option of StringTie version 1.2.2 (Pertea et al., 2016). Transcripts with FPKM values greater than 0 in at least one sample were retained.

### Comparisons Between LincRNAs and Protein-Coding Transcripts

Detailed information on 45,788 protein-coding transcripts were extracted from the pig reference genome annotation file (*Sus scrofa* 11.1^3^). Detailed information on 252 putative lincRNAs were extracted from the unique transcriptome file. Then, we compared the transcript length, exon length and exon number between lincRNAs and protein-coding transcripts.

### Analysis of Differentially Expressed Genes and DEPTGs

We used the htseq-count tool to count how many aligned reads of each gene overlap with their exons (Anders et al., 2015). Then, through the DESeq2 tool (a count-based technique) in the R packages (version 3.4.3) to perform differential expression analysis of gene-level between fat and lean group using these counts (Love et al., 2014). The gene with an FDR-adjusted *P*-value less than 0.05 will be considered as a differentially expressed gene between the two groups (Benjamini et al., 2001). If the expression level between each pair of lincRNA and the protein-coding gene was significantly correlated (*P*-value < 0.05), we regarded a protein-coding gene as a PTG of lincRNA. Meanwhile, if the potential target protein-coding gene was differentially expressed, we regarded it as a DEPTG of lincRNA.

### Gene Ontology (GO) and Pathway Analysis of Adjacent Genes of LincRNAs

The position information of lincRNAs were extracted from the unique transcriptome file. Then, the neighboring genes (<100 kb) of lincRNAs were obtained by BEDTools version 2.17.0 (Quinlan and Hall, 2010). Due to the limited annotation of the pig genome, we need to use Ensembl to convert the genes into human homolog genes (Kersey et al., 2014), and then used DAVID to perform GO enrichment and KEGG pathway analysis (Huang da et al., 2009). The *P*-value less than 0.05 was considered statistically significant.

### QTL Mapping Analysis of DELs

Firstly, the position information of DELs were obtained from the unique transcriptome file. Then, we downloaded the pig QTL database^6^ from the animal QTL database. After that, we used BEDTools version 2.17.0 tool for QTL mapping of DELs (Quinlan and Hall, 2010).

### Correlation Analysis of DEL Genes and Their Adjacent Genes

The position information of the DEL genes were obtained from the unique transcriptome file. Then, we identified the neighboring protein-coding genes (<100 kb) of each DEL gene using the BEDTools version 2.17.0 tool (Quinlan and Hall, 2010). The Pearson correlation coefficient were calculated based on the expression level of each pair of DEL gene and protein-coding gene (Salleh et al., 2015).

### Correlation Validation Between LincRNA Genes and Their PTGs Through the Real-Time Quantitative Polymerase Chain Reaction (RT-qPCR)

We used 11 large white pigs with the BFT ranging from 18 to 22 mm for RT-qPCR verification. Total RNA was extracted from adipose tissue by using Trizol reagent (Invitrogen, Life Technologies, Foster City, CA, United States) from the PSCs according to the manufacturer’s instructions. The purity and concentration of total RNA were measured by a micro-spectrophotometer (Thermo, NanoDrop 2000, United States) at 260 and 280 nm. Ratios of absorption (260/280 nm) of all samples were between 1.8 and 2.0. cDNA synthesis for lincRNA genes and PTGs detection was performed using RevertAid First Strand cDNA Synthesis Kit (Thermo, Wuhan, Cat#k1622). We used approximately 1 μg of total RNA for cDNA synthesis. RT-qPCR for lincRNA genes and PTGs detection was performed with SYBR Green (CWBIO, Beijing, China, CW0957) in Roche LightCyler 480 system (Roche, Mannheinm, Germany) according to the manufacturer’s instructions. Seven pairs of RT-qPCR primers were designed using the Primer 5 program (Supplementary Table S1). The 18S rRNA was used as an endogenous control gene. The RT-qPCR data were analyzed using the 2^-ΔΔCT^ method.

## Results

### Identification of LincRNAs

**FIGURE 1:**
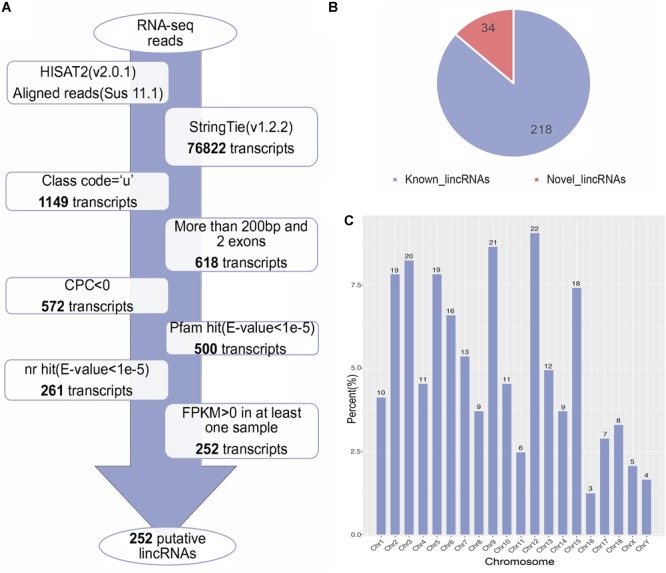
**(A)** The pipeline for the identification of putative lincRNAs in this study. The frames in the direction of the arrow show the filtering process and the number of screened transcripts. **(B)** The number of known and novel lincRNAs. **(C)** The chromosome distribution of putative lincRNAs.

We used the published RNA-seq data from ILW pigs to identify lincRNAs (Zambonelli et al., 2016). These RNA-seq data were divided into two groups according to the BFT EBV (Zambonelli et al., 2016). The numbers of raw reads per sample were between 161.88 and 249.33 million. After removing adapter and low quality reads by using fastp software (Chen S. et al., 2018), approximately 152.81 to 236.66 million reads were mapped to the whole genome of *Sus scrofa* (11.1) (Table 1). Then, we reconstructed the transcriptome of each sample separately. Next, the transcripts of all samples were merged into a unique transcriptome. At this point, we obtained 76,822 transcripts, of which 1,149 were intergenic transcripts. We identified 252 putative lincRNAs from 1,149 transcripts based on the pipeline shown in Figure 1A, in which 34 did not overlap with the currently annotated coding or non-coding transcripts (Figure 1B). These putative lincRNAs were distributed in all chromosomes (Figure 1C).

### Characterization of LincRNAs

**FIGURE 2:**
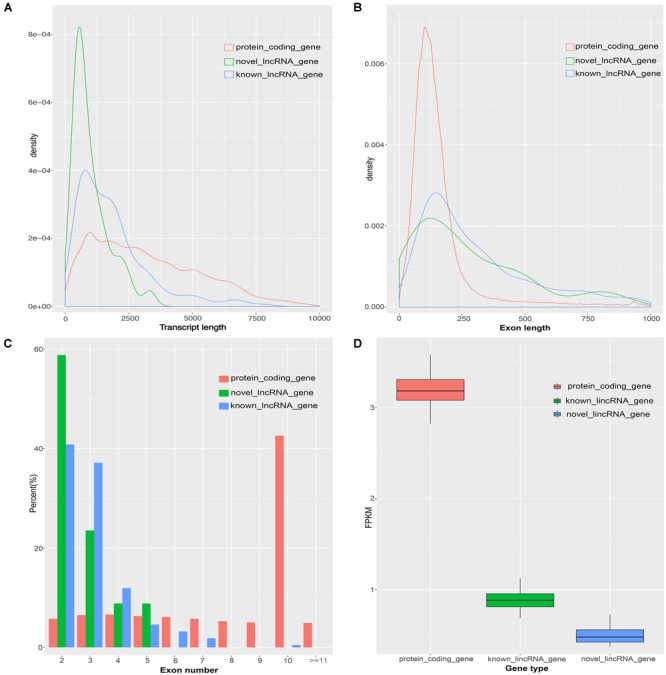
Comparison of the characteristics of protein-coding genes, novel lincRNA genes and known lincRNA genes. **(A)** Comparison of transcript length. **(B)** Comparison of exon length. **(C)** Comparison of exon number. **(D)** Comparison of the expression level.

Previous studies found many differences between lincRNAs and protein-coding transcripts (Derrien et al., 2012; Liu et al., 2017). Based on the assembled transcriptome, we compared the characteristics of novel lincRNAs, known lincRNAs, and protein-coding transcripts. A total of 45,788 protein-coding transcripts corresponding to 22,342 genes in the pig annotation in Ensembl database and 12,103 known lincRNA transcripts encoded by 7,381 lincRNA genes in the pig lincRNA annotation in ALDB were observed (Li et al., 2015). The average transcript length of the protein-coding transcripts (3,285 bp) was longer than the novel lincRNA transcripts (1,011 bp) and the known lincRNA transcripts (1,891 bp) (Figure 2A). At the same time, the average exon length of the protein-coding transcripts (283 bp) was shorter than those of novel lincRNA transcripts (378 bp) and the known lincRNA transcripts (628 bp) (Figure 2B). In addition, the novel lincRNA transcripts (2.7) and the known lincRNA transcripts (3.0) had similar average exon number, but all were less than the protein-coding transcripts (11.6) (Figure 2C). In addition, the average expression level of protein-coding transcripts in the samples (3.18 FPKM) was higher than those of the novel lincRNA transcripts (0.51 FPKM) and the known lincRNA transcripts (0.90 FPKM) (Figure 2D). Our results are consistent with previous reports that the pig lncRNA genes have shorter transcript lengths, longer exon length, fewer exon number, and lower expression level than protein-coding genes (Zhou et al., 2014; Li et al., 2016; Tang et al., 2017).

### Differential Expression Analysis of LincRNA Genes and Protein-Coding Genes

**FIGURE 3:**
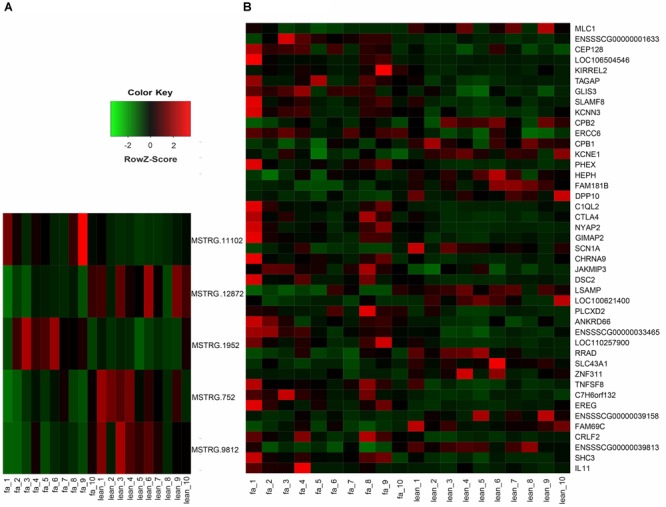
**(A)** Heat map of five DEL genes between fat and lean samples. **(B)** Heat map of 43 differentially expressed protein-coding genes between fat and lean samples. Red represents an increase in expression, while green reduces expression.

To investigate the function of lincRNAs, we performed differential expression analysis on fat and lean samples based on expression levels. In both groups, we identified five DEL genes. Compared with lean pigs, two lincRNA genes (MSTRG.11102 and MSTRG.1952) were upregulated and three (MSTRG.12872, MSTRG.752, and MSTRG.9812) were downregulated in fat pigs (Figure 3A). In addition, we identified 43 differentially expressed protein-coding genes with 27 upregulations and 16 downregulations in fat pigs (Figure 3B).

### Functional Enrichment Analysis of Adjacent Genes of LincRNAs

**FIGURE 4:**
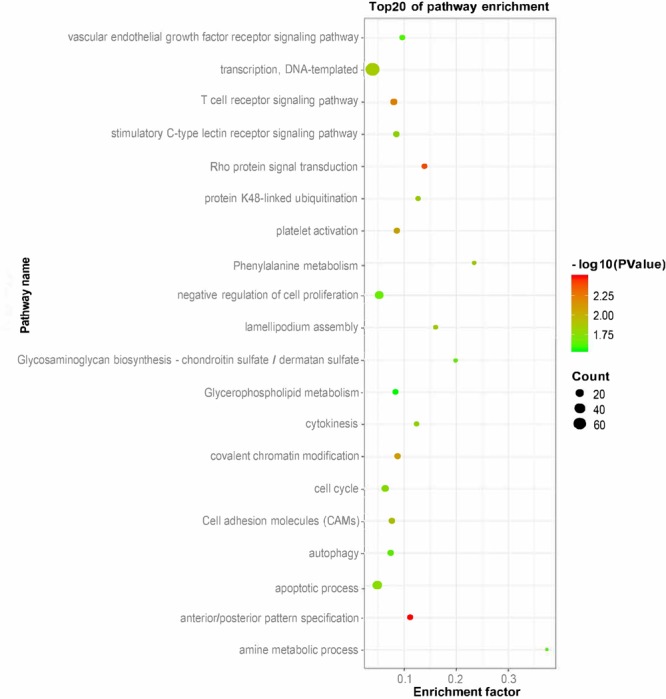
Gene ontology and pathway analysis of adjacent genes of lincRNAs.

To explore the function of putative lincRNAs, we performed functional enrichment analysis of genes near the lincRNAs (<100 kb) with DAVID (Supplementary Table S2). The DAVID results showed that the adjacent genes of lincRNAs significantly participated in 25 biological processes (apoptotic process, amine metabolic process, negative regulation of cell proliferation, and protein K48-linked ubiquitination) and six pathways (phenylalanine metabolism, cell adhesion molecules, and glycosaminoglycan biosynthesis-chondroitin sulfate/dermatan sulfate) (*P* < 0.05). We found that the neighboring genes of lincRNAs were involved in lipid metabolisms, such as glycerophospholipid metabolism (Figure 4).

### Functional Prediction of DELs

**FIGURE 5:**
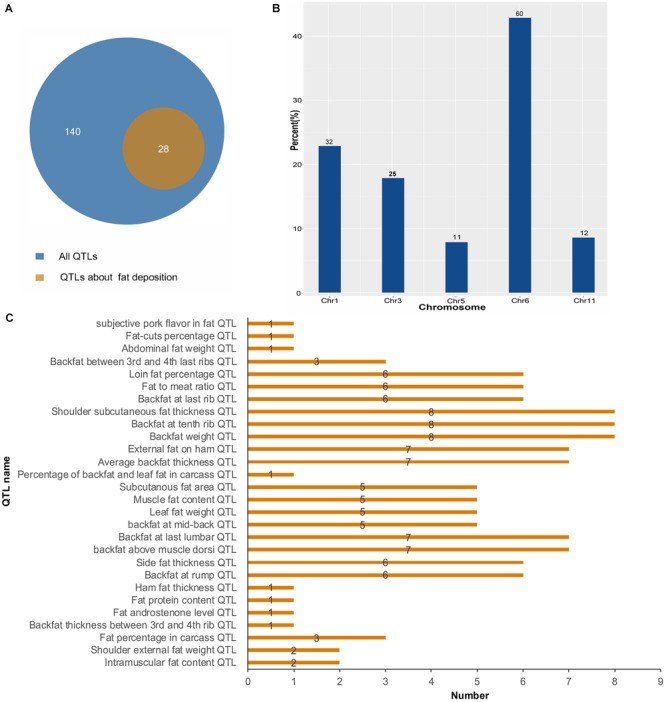
Quantitative trait locus mapping analysis of DELs. **(A)** The number distribution of different QTLs. **(B)** The quantitative distribution of DELs which were located in QTLs associated with fat deposition. **(C)** The chromosome distribution of all QTLs.

To further investigate the function of lincRNAs, we performed QTL mapping analysis on DELs (Supplementary Table S3). The result showed that 10 DELs were located in 140 QTLs, wherein 28 QTLs were associated with fat deposition (Figure 5A). Through the distribution of DELs in QTLs, we found that all 10 DELs were located in QTLs related to fat deposition, and most of the DELs were located in QTLs associated with backfat (Figure 5B). For example, eight DELs were located in the QTLs of the backfat weight QTL and shoulder subcutaneous fat thickness QTL. Seven DELs were located in the average BFT QTL and backfat at the last lumbar QTL. Simultaneously, we found that 140 QTLs were distributed on 1, 3, 5, 6, and 11 chromosomes (Figure 5C). In summary, we found that DELs mainly located in QTLs related to fat depositions, such as shoulder subcutaneous fat thickness, backfat weight, and average BFT.

### Correlation Analysis Between DEL Genes and Its Adjacent Genes

Previous studies indicated that lincRNAs can affect the expression of their neighboring genes (Fatica and Bozzoni, 2014; Taylor et al., 2015; Engreitz et al., 2016). According to the expression level, we calculated the Pearson correlation coefficient of DEL genes and their nearby protein-coding genes (<100 kb), including three DEL genes and six protein-coding genes (Table 2). The results showed that the expression levels of three DEL genes were significantly positively correlated with five adjacent protein-coding genes and significantly negatively correlated with one adjacent protein-coding gene. Compared with lean pigs, MSTRG.11102 and MSTRG.1952 were upregulated in fat pigs, whereas MSTRG.9812 was downregulated. The expression of five adjacent protein-coding genes was upregulated, and one was downregulated by three DEL genes. Then, the function of each DEL gene was speculated according to the functions of their nearby protein-coding genes. Among them, the expression level of DEL gene (MSTRG.1952) was significantly positively correlated with the nearby protein-coding gene MEDAG (also known as MEDA-4). The expression level of DEL gene (MSTRG.9812) was significantly negatively correlated with the nearby protein-coding gene SPTBN1.

**Table 2:** The correlation between DEL genes and its adjacent genes.

DEL	Neighboring protein-coding gene	Pearson correlation coefficient	*P*-value
MSTRG.9812	ENSSSCG00000026559 **(EML6)**	0.611753737	0.004152
	ENSSSCG00000040786 **(SPTBN1)**	–0.519505779	0.0189
MSTRG.11102	ENSSSCG00000037425 **(NFAM1)**	0.761190956	0.00009702
MSTRG.1952	ENSSSCG00000009330 **(ALOX5AP)**	0.515617225	0.01997
	ENSSSCG00000009331 **(MEDAG)**	0.515617225	0.01997
	ENSSSCG00000009332 **(TEX26)**	0.515617225	0.01997

### Expression Regulation Analysis of DEL Genes and Their DEPTGs

**FIGURE 6:**
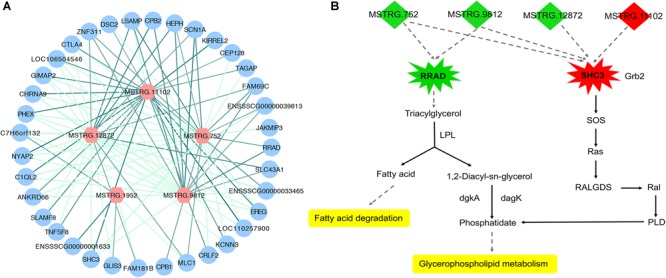
Expression regulation analysis of DEL genes and their DEPTGs. **(A)** Co-expression network of DELs and DEPTGs.DELs are indicated in red hexagons;DEPTGs are indicated in blue circles; light green edge represent DELs downregulate DEPTGs; dark green edge represent DELs upregulate DEPTGs. **(B)** Potential pathways for DELs and DEPTGs. red indicates that the genes are upregulated in fat pigs, green indicates downregulation in fat pigs, and yellow indicates potential pathways for DELs and DEPTGs; quadrilaterals represent DELs, irregular polygons represent DEPTGs, and dash lines indicate predict interactions.

To investigate the function of the DEL genes, we analyzed the expression regulation relationship between DEL genes and their DEPTGs (Supplementary Table S4) and constructed the co-expression network by using Cytoscape_3.6.1 (Figure 6A). Based on the expression level, we found that five DEL genes and 36 DEPTGs were significantly correlated (Figure 6A), among which 32 of 36 DEPTGs were regulated by two or more DEL genes. Thus, we speculated that the regulatory mechanisms among DEL genes and their DEPTGs were complicated. In fat pigs, the expression levels of MSTRG.752, MSTRG.9812, MSTRG.12872, and RRAD were downregulated, and the expression levels of MSTRG.11102 and SHC3 were upregulated. Furthermore, the expression level of RRAD was significantly positively correlated with MSTRG.752 and MSTRG.9812. The expression level of SHC3 was significantly negatively correlated with MSTRG.752, MSTRG.9812, and MSTRG.12872 and significantly positively correlated with MSTRG.11102.

### Correlation Verification Between LincRNA Genes and Their PTGs

**FIGURE 7:**
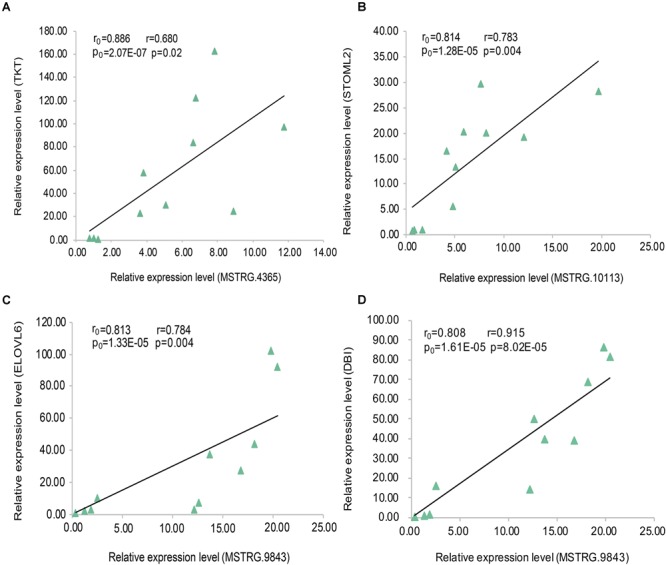
Linear regression of lincRNA and PTG expression. The r_0_ and p_0_ represent the Pearson correlation coefficient and *P*-value of each pair of lincRNA and PTG in 20 samples, respectively;The r and p represent verification in 11 samples. **(A)** MSTRG.4365 vs. TKT; **(B)** MSTRG.10113 vs. STOML2; **(C)** MSTRG.9843 vs. ELOVL6; **(D)** MSTRG.9843 vs. DBI.

In the data analysis results of 20 samples (10 fat samples and 10 lean samples), we randomly selected three lincRNA genes (Supplementary Table S5) and their PTGs with significant positive correlation based on the expression level (MSTRG.4365 vs. TKT; MSTRG.10113 vs. STOML2; MSTRG.9843 vs. ELOVL6 and DBI). The correlation coefficients were all greater than 0.80, and the *P*-value were less than 0.01. To verify these results, we conducted the RT-qPCR experiment in 11 samples and performed linear regression analysis on the results. The four pairs of lincRNA genes and their PTGs were significantly positive correlated based on the expression level, with correlation coefficients greater than 0.65 and *P-* value less than 0.05. The experimental results of RT-qPCR showed that the results of the two datasets have good consistency, thereby further improving our research reliability (Figure 7).

## Discussion

A large number of lincRNAs are present in the mammalian genomes (Carninci et al., 2005; Cabili et al., 2011). A small number of lincRNAs have been revealed in pigs; these lincRNAs play a key role in biological processes (Li et al., 2016; Che et al., 2018). In Zou’s study, some lincRNAs in longissimus dorsi muscle of Laiwu pigs may be involved in intramuscular fat-related biological processes, such as oxidative metabolism, lipid metabolism, and adipogenesis (Zou et al., 2018). Zou et al. (2018) provided a valuable resource for further study of lipid metabolism in pigs. However, a large number of lincRNAs are still unknown. In particular, many lincRNAs associated with subcutaneous fat development in pigs have not been identified. In this study, based on the published RNA-seq data, we performed a comprehensive identification and analysis of lincRNAs in subcutaneous adipose tissue of ILW pigs. We identified 252 candidate lincRNAs by using our laboratory’s previous research methods, wherein 34 of which were novel lincRNAs (Zou et al., 2017b), further enriching the annotation of lincRNAs and providing new insights into the study of lincRNAs in pigs.

In this study, lincRNAs have shorter lengths, fewer exons, and lower expression levels than protein-coding transcripts, consistent with previous reports in pigs and increases the reliability of our study (Zhou et al., 2014; Li et al., 2016; Tang et al., 2017). Our research is difficult because lincRNAs are largely unknown. LincRNA has several ways to regulate gene expression, such as *cis*- and *trans*-regulation (Yap et al., 2010; Trimarchi et al., 2014; Carmona et al., 2018). At present, *cis*-regulation is an important method for studying lincRNA function. We mainly looked at neighboring genes as PTGs in this work. Previous studies investigated the function of lincRNAs by genes that are adjacent to the lincRNAs (<100 kb) or have expression correlation with lincRNAs (Hong et al., 2018; Zhang et al., 2018). In this article, we identified 252 candidate lincRNAs between fat and lean samples. According to GO and pathway analysis, we found that the adjacent genes of lincRNAs (<100 kb) were involved in glycosaminoglycan biosynthetic process, glycerophospholipid metabolism, and glycosaminoglycan catabolic process. Among them, glycerophospholipid metabolism is closely related to fat deposition (Ecker and Liebisch, 2014; Jiang et al., 2018). Thus, we speculate that lincRNAs may have a regulatory role in subcutaneous fat development. The QTL mapping analysis of DELs further improved our speculation.

To determine the regulatory mechanisms of lincRNAs, we summarized the expression relationships of five DEL genes and their adjacent protein-coding genes (<100 kb). The expression level of lincRNA gene (MSTRG.1952) and mesenteric estrogen-dependent adipose gene (MEDAG, also known as MEDA-4) were significantly upregulated in fat pigs compared with lean pigs. MEDAG has previously been identified as a novel adipogenic gene in mouse and human that promotes lipid accumulation in adipocytes (Zhang et al., 2012). From this finding, we hypothesized that MSTRG.1952 may promote fat deposition by positively regulating the MEDAG expression in fat pigs. At the same time, high expression of lincRNA gene (MSTRG.1952) significantly upregulated the expression of arachidonate 5-lipoxygenase-activating protein (ALOX5AP) in fat pigs. Previous studies confirmed that ALOX5AP overexpression in mouse adipose tissue leads to lipoxin A4 (LXA4) production and diet-induced obesity (Mehrabian et al., 2005; Horrillo et al., 2010; Elias et al., 2016). In addition, obese subjects have significantly higher ALOX5AP expression in subcutaneous adipose tissue than lean subjects (Kaaman et al., 2006). Therefore, we hypothesized that MSTRG.1952 promotes the subcutaneous fat deposition by positively regulating the ALOX5AP expression in fat pigs.

Based on the expression level, we analyzed the expression regulation relationship between DEL genes and their DEPTGs. The results showed that five DEL genes and 36 DEPTGs were significantly correlated. According to previous studies (Pelicci et al., 2002; Ilany et al., 2006) and KEGG pathway database^7^, we found that some DEPTGs may be involved in lipid metabolism, including Ras-related glycolysis inhibitor and calcium channel regulator (RRAD) and SHC adaptor protein 3 (SHC3). Therefore, we constructed a potential pathway map of key genes and related DELs affecting lipid metabolism between fat and lean pigs (Figure 6B). Previous reports indicated a certain correlation between the expression level of RAD (also known as RRAD) and obesity (Ilany et al., 2006). The expression levels of lincRNA genes (MSTRG.752 and MSTRG.9812) and RRAD were significantly downregulated in fat pigs compared with lean pigs. RRAD overexpression can promote the increase in lipoprotein lipase (LPL) protein level, and LPL hydrolyzes triglycerides in lipoproteins into free fatty acids and monoacylglycerol molecule, thereby decreasing the triglyceride levels and affecting lipid metabolism (Ilany et al., 2006). Therefore, we speculated that MSTRG.752 and MSTRG.9812 promote fat deposition by reducing the RRAD expression in fat pigs. In addition, compared with lean pigs, the expression level of lincRNA gene (MSTRG.11102) and SHC3 were significantly upregulated in fat pigs, and the expression levels of lincRNA genes (MSTRG.752, MSTRG.9812, and MSTRG.12872) were significantly downregulated. Literature showed that Rai (SHC3) overexpression can induce Ras activation (Pelicci et al., 2002). Based on the KEGG pathway database^7^, we found that Ras may further affect glycerophospholipid metabolism. Thus, we speculated that MSTRG.752, MSTRG.9812, MSTRG.12872, and MSTRG.11102 promote fat deposition by increasing the SHC3 expression in fat pigs. Collectively, we speculated that four DEL genes may affect subcutaneous fat development by regulating the expression of RRAD and SHC3.

In this study, we identified and analyzed the lincRNAs in the subcutaneous adipose tissue of pigs and found that some lincRNAs may affect subcutaneous fat deposition, especially DEL genes, thereby resulting in the difference in BFT between fat and lean pigs. However, the specific regulation mechanism of lincRNAs on subcutaneous fat development is still unclear, and further studies are needed. Given that pig lincRNAs are largely unknown, our research provided valuable resources. This work also provided an ideal candidate for future studies of the function of lincRNAs in the subcutaneous fat development.

## Data Availability

Publicly available datasets were analyzed in this study. This data can be found here: https://www.ncbi.nlm.nih.gov/sra?LinkName=bioproject_sra_all&from_uid=281483.

## Author Contributions

CL conceived and designed this study. GS, LC, and GC analyzed the data with the help of CZ and CF. GS wrote the paper with the help of CZ and CF. RT-qPCR experiment was completed with the help of JL and ML. All authors reviewed the final manuscript.

## Conflict of Interest Statement

The authors declare that the research was conducted in the absence of any commercial or financial relationships that could be construed as a potential conflict of interest.

## Footnotes

1 http://res.xaut.edu.cn/aldb/download.jsp

2 http://www.noncode.org/download.php

3 http://ftp.ensemblorg.ebi.ac.uk/pub/release-93/gtf/sus_scrofa/

4 https://www.ebi.ac.uk/Tools/st/emboss_transeq/

5 http://pfam.xfam.org/search

6 https://www.animalgenome.org/cgi-bin/QTLdb/SS/index

7 https://www.genome.jp/kegg/pathway.html

## Supplementary Material

TABLE S1 The information of seven pairs of RT-qPCR primers.

TABLE S2 Gene ontology and pathway analysis of adjacent genes of lincRNAs.

TABLE S3 Quantitative trait locus mapping analysis on DELs.

TABLE S4 The expression regulation relationship between DEL genes and their DEPTGs.

TABLE S5 Sequence information of lincRNAs used in RT-qPCR.

## Abbreviations

ALDB: the domestic-animal lncRNA database
BFT: backfat thickness
CPC: coding potential calculator
DAVID: the database for annotation, visualization and integrated discovery
DELs: differentially expressed lincRNAs
DEPTGs: differentially expressed potential target genes
EBV: estimated breeding value
FPKM: fragments per kilobase of transcript per million mapped reads
GEO: Gene Expression Omnibus
GTF: gene transfer format
GO: gene ontology
ILW: Italian large white
KEGG: kyoto encyclopedia of genes and genomes
LincRNAs: long intergenic non-coding RNAs
LncRNA: long non-coding RNA
NCBI: National Center for Biotechnology Information
NR: non-redundant
PTG: potential target gene
QTL: quantitative trait locus
RT-qPCR: real-time quantitative polymerase chain reaction
