# Supplementary material for: Identification and Functional Prediction of Long Intergenic Non-coding RNAs Related to Subcutaneous Adipose Development in Pigs
Source: Front Genet. 2019 Mar 4;10:160. doi: 10.3389/fgene.2019.00160 (PMC6409335; doi:10.3389/fgene.2019.00160)
Supplement: TABLE S5 — Sequence information of lincRNAs used in RT-qPCR. [file Table_5.DOCX]

MSTRG.4365

CGGGAAGCATTTATTTAACGGCAACCGTGATCCGTAAGACATTCAAGGAAAAAAAATGTGTTTTTCCACTTTGGGAAATAGTCAAAGCTTTTATATTCCACCAAAAATAGAAGTGGAATTCTCGAAAGCAAAAATAGGTGGGCTTATCCATGTATGCAACTCTAACGAACCACCAGTCTGCAATGCTCTCAGCTCTCTGGAATGCGTGCCTGCGTTTCCGAGGCTGCGGACAGACAGGCGGCGGGAGGAACCCGGGACCTGCTCTGGGGCTGTGCCAGCTCGGCCGCCTGGACCCTCCCCGGTCCCGCTTCTTCACAGGCCTGCGTTGCTGGTTGGTAACGCTGTCACAGCCCCCATGGTGGCGGGTTTGCACAGGCAGAGCCTTCCCCTGTTAGGACCCTGATGGTTTAAAACACCGAGAGCCACATCCGTGTACGGCTGGTGGCGGGGCTGGGTCTCTGCATATACCCTCGGCAGGCAGAAGCAGTGACTTACATCTCTGTGTAATATTTATTACGCTTAGCTTACAGCTGGTTTTACATACTTTGTCGTAACAATGTCGCTGCTTATCTCCATGAGACGTGACTTGAGACCTGCTCTCTGAGGAGGACAGTGAGGCCCAGAGAGCCAGCAGCCCGCCTGAGGAGTGGTCCATCCCCCACCGGCCACGGCTGCCTTCTCCCGAGGGTGTCCCTTCCTCCAGGCACTGACTTTGAATGGTGGCCTCATCCAGCCGCGGTCCTCCAGCTCCGAGCTGCGCTCTCGCACCCGGGCCCCTGAATGGATTCATGTGTGTATGCAGTGCTAAATACGCTCACACACAGCATTGCACAGCTACACGCGCCTCAGCCCCCCGTTTTCAGGCTCAGTCACATGGCAGGCTTCTCAGCCAACATTTAATTCCCAAGGTTTAATTCCCAAGGTTCACCCACGCTGGGTGTGGCAGTGGCTGAATCGTTGAACAGCCTCTCGGTCACGTCCCCCACGTGCGGACAGCATGTGGGGAGCCCCAGGCGTCGGGGGAGCCCCAGGCGTCTCGTGCACCCCAGGGAACGGATGGTGGGATCTGTGAGCACGGCCAGGGCGCACGTCTGCTGTCTGCCTCTGGGCCACACGTGGGAGGCTTCGTCCTGCTAAACACAATTATTCCGGAATACAAACAGTCTTGTAATACAAGGCATTTCCCAAGGGGACACGCTTTCCAAAGGGGTCGCAAATTATTGTCCTGCTGGCCGTGATCCAGAATTCTGGGGCCCACGCCCTCTCCATCGCTTGCTCTCTATTGCCAGCTTTCATCTTTGCCGGTATGGTGGCTGTAGCCTAGTATTATTTTGACTCAAGTCTGCGTTTCCGTGTCATGGGTGAGGTCTTTCCACGCATTTCAGGCATACGTTTGCTCCTCTGCTCACGCCTGCTCGGGTGTTCCCCACTTCTCGTGAGCATGTACCCCTTCCGTATTGGACGTGTGGTTCTTTCTATGCCTGAATACAAGTCCCCTACTAGTTACACGGGCTCGCCTGGGCACAGGACACACTGCCGAAGGCACCGGAAACCATCAGATTTGGGGATGCCCTGGCTCTGCCTGCACCCCGTGAGTCTCATTTCAGGCTGGCGTCCCTGATAGGAGTACACGGCAGACTGTCCTGACCGCCTCTGGGTTTCAACGTGTCAGGTCGAGGAGCAGAGGGAGCCTCAGGACCGGCCCCTGAGTCCGTAGTCCTCCTCTGGGCAGCGGCGGGGAGGTGTGTGTGTGCTGGGGGACCCCCAGGGCTGCTCCGGGCCTGGTGGCGACCAGAGGGATTCACCGTGGGGGCCGGGAACCTCAACAAGCCCAGCAGACTCGGAGCAAGGTGTCCACCCCAGGAAGAGCTGACCGAAGGGTTAGTGGCATCCGGCTGGTATCGCTCCAGTCACGCTCTCGCCCGGGCCCCGCGCTGCAGGCCGCCGGCGGGTGTGCGTGCGCCGGGGTCTCCTGGCTCCGTGCACCTGCCGGGCTCCCCCTCCCCAGCCGCTCCCGGGCCGGGCGGCCGGCCCCCCGCGGACCTCCGGCCGGGCTGGAGGAGGCGTGCGCGCGCGCGCCCGAGCTGCGTG

MSTRG.10113

TAAATTTAATAATATATTTTATTTACCTTAACACATCTAAAACATAATTTCCAGAGGCAATCAATATAAAATTATTAGAAAGATATTTGGCATTTTTTTCAACGTCTTTGAAATCAGGTGTTTCTTGTGCACATTCGCACATCGCAGTTGGGACAGGCTGAGGCCACTGTGCTGGACGGTGCAGAGCCAGAGCAGACGGGCTCCTTGGCCTCTCCCTCGAGGGCTGTTCTCTTTCCACTCCCTGCTCCCTGCCAGTGGGAGCGGTCAGCAATCCCCTTGTTCCCCAAGAGTTAAAACAAGGGGTAAATCAGCAGGTTTCTGCCCCAAACGTCTCCGAGTGGAGGCTTTGACCTGACTGAGATTATTGGCAACCCCAGGGCCAGTTAACTCAAATGTCTACACGACAGGCGGGATGTCATCAGCTCCCACCCGGAGCAGCAGCTGCCTCCACCCATCTCTGACTTTGCTCTGCACTGTATGAGGGTCGAGGCCCCTCGGGCTGTGCCCAAGGCCCTCCTGAGCCCACCGGGGCCTGAGGGTGGCGGGCAGGACACAGACCTGAAACCAAACCCCATCTTGCAGATGCTACCAAGCAGTTCACAGTGCCTTCTCAAGTCCGTGGTCTTCTGTAGGCTCCCTGGGAGTCCGTTCAGGGCATCCTCTGTGAGCTAAAGGGTCTGGGTGGCCCTAAGGGTGGGGAGAAGATCACGTGGCATTAGACCTTGACTCAACATTAGAACAAGGAGCCCCTCAGCCTGCCAGACGCTGGCACCTACCCACCCTCTTCTCCAGCCCAGTGTCACACCTGGGCCTGTGCTCATCCATTTTATTAGTTAACATATATTAATTTCACTTACTGCCTGAAACAAAACCTTCCAACATCAGTCTATAATCCTTGATAAAATTAGAAATTATGAGGAGTTCCCTGGTGGCCTGGTGGTTAAAGGATCCCGCATTGTCACTGCTATGGCTCTGGTGACTTCTATGGCACAGGTTAGATCCCTGGCCTGGGACCTTCTACGTGCTGTAGGCATGGCCAAAAAAATTACAAATGACAAGCATTCGGCAGCAATCTCGTTCTCCTGATAGAACGATCTGACAAATGTGATTTTTCTGATTTACAAAATGGAGGGTAATTGTTCAGTAAACATGGCTGTACACTGATTACGTGACCTTTCAGGGAAGGGTTACACACGCTTTTCTTGGGTATCTGTGATGCTCCCCCAGAACTGACACATTTTCCCCGGCACACTCGCCTTCAGCCTGCCCCCAAGAGCTGTTATGAGAGGCAGAAGGACCTGTGGGTAAGAGGGGTGGGCCACGCTCAGACCCTACAGCACCTTAACCGGCTCGGGGACCAGGCCTAGGGGAAGGCCTTCCAGGTAGGAGCCCTTGGGGCAGCGGAGGTGAACTCCACGTCCCGGCCGCGCATCCAGACTGGACCTATGGGGCCACTGGAGGACTCTGTAGCCGTGGGAGGATAGAGCCCTGCCCCCAGAGCACCTACAGGGGCTCAGCCGAGACGGGCTCCGGCTGCAAGAGGCCTCACAAGAACAACTGAGAGGGTCATGGGTCTGAAGGACGCGAGGAGGAGGGGAGAAGGGGGGCGACGACGGGGGGCGGAGGTCGCCAAGGGGTGAGGGGCTGAGACCCACCTTTGCCTGCTGTAAGACACACCTGTGTGCCTCACCTGCCATCCCTGGACTTTCTGAGCAGCTGCCCAGAGAGACCGGACCCAGACACAGCCCCAGGGTCCTGCAGCACAGCTGTCCTGCCACTGGCACTCGCCTGACAGCCAGGCCTGCTTCACCCATGAAGAGAGCAGGACAGAGTGCAAAAGGGGAGAGGCCCGATGCTCCCCCAGCGCTGGAAGGCTTCGGCTTGCACTGCCTCCCCCGGAGGCCCTAGCAGCACAAGGGACAACCCAGCCCCTAAGAAGCCTCCTTCTCCCGCTGGTGGAAAACAGGCTGCAGAGGAGGCCCACTCCCAGGCCTGCTGCGAGGGGCGGGGGACAGGGCCTCCCACCCTCCCTGGCATCAGGAGCTTCGCGGTGAGTGCGCCTGCCCACTTCAGCCATATTTTTCCACATTTGGATTTCATCCTGGCCAAGCTGGAATCCCTGTTCTGAATGAAGGAAACTGTTGTTGTTTTTTTAGGGCCGCACCCGCAGCATGTGGAGGGTCCCAGGCTAGGGGTCCGTTCGGAGCTGCAGCTGCCGGCCTACACCTCAGCCACAGCAACGCTGGATCCGAGCTGCATCTGTGACCTACACCACAGCTCATGGTGTTGGGGAGACGGCTCGGATCGTCCGTGTCGGAAAATGAGACACATGAGCATGGGGAGGTCTCGACAAGGCGCTTTCCTTCTGCAGAAGGGCGAGGGGGCTCAGAAAGCGCGCCAAGAAGACCCTCCCTGTTTCTCCCTAATGCAGGCGACGGACCTGTCCCCTTCCCATGGGTCGGGTCAGGGCGCACTTTCTTCTCGGTGGGCTCATCTGAAACAAATTGTTACCAGGAGAAGAGGGAAAGAGGAGGGGGTCGCAGGAGGGCGGTTCAGCTGAAACGGGAGCAAAATGGGGTCACAAGATTTCCTTCAAAGGAAAACACATTACTTTCCACGACGGCAACGCAGGACCCTTAACCCACTGAGCGAGGCCAGGGATTGAACCTGCAACCTCGTGGTTCCTAGTCGGATTCGTTTCTGCTGCGCCATGAGGGGAACTCCAGGAAATTGTTTCATTTCCGGAAATCCTGTTTTTCACCTTTTATCTCCTTTCAAGATAAATAAGTTTGCTGAGCCAGAGCGTCACTGGCCTTTCAGACATGTTTTTATTGTGCGCGGCCCTCCTTGAGCACAGGAGAGATGCAGGGACTCAGGTCTGAGCAGAAGGAAGTTGAGTGCTGGACAGGAGTCAGCTGGGAGCAGGTGGTGACAAGACCTCTCTGCGCAGGCCATAGGCCTGTCACCAGGAGCCCTGCCACCTGCCTCCTCAGGCCCCCTTGGCACCTACTTTTAGCCCTTCCTTGCTTTTAGGCTCAGGATCCTGAGCCCCTGACCTGTTCAGGTAAAACCCTGGGGAGCTCAGGGCTGCTTCTGTTCGGGGCTCCGTTTCCTCCCGCCACCTTTAGAGCCCTGGCTAGATCCAAAGACACCCACAGAGACAGCAAACAATCCTCAGAGCCAGGCTTTGATCCAAAACAACCAATCCCGAGCCTACACTCCTGACTTCTCCCTTATCAGACTCGCACACCACACCAGCGTCCCCCGCCCTGAATCACCCCAGGGGCAGGTATTGGACAGCTAGAGACCACCGCTACAGCGCAGCCTGCTGAGATTTTTCAAACGATAGGACACCAAGCTTTCTCAGCGGACCTGCCCGCCTCGCCCATTCCTTCCCATGTAAACCCCAATAAAGGCTCTGGGCTTCGCTTTCCTCCCTGCTTTTGCCTCCTGGCCAACCTGGTGCTTCACTGTGCCGCCCTGCAGGGTGTGTCCTGCCTTCTGTTCCTAGAGACCTGTGGGTACACATGTCTTCCTTCATTTCCGAGTCTGCGTGAACTACCATCCCCTGAAGAACACAAACCACAGGTACGTTTTCACACAGATTGCTGACGCCCAAGCACAGCGGTGGCCTTCAGAGGGGCTCTCTGTTCCAGTTCATTCAAAATGTTACTGCCCTAGGAGTTCCCGCCGTGGCACAGTGGTTAACGAATCCGACTAGGAACCATGAGGTTGCGGGTTCAGTCCCTGCCCTTGCTCAGTGGGTTAACGATCCGGCATTGCCGTGAGCTGTGGTGTAGGTTGCAGACGCGGCTCGGATCCTGCGTTGCTGTGGCTCTGGCGTACAGCTCTGGCTGTAGCTCCGATTCGACCCCTAGCCTGGGAACCTCCATATGCCGCGGGAGCGGCCCAAAGAAATAGCAAAAAGACAAAAAAAAAAAAACAAAAAAAAAAACCAAAGTGTTACTGCCCTAGGGCACTGTCTAGGAAGCCGAAAGTGGCCTAGGAAATTTATCTCTTTGAAAACTCATATTTATTTTATTTTATTTTATCTTGGCTGTGCCTGTGGCATGCAGAGGTTCCAGCACCAGGGATCAAACCACACCAAAGACCTGAGCCGCTGCAGTGACAACACCAGGGGGCTCTTCAAAAATGATTTTTAAATTGGCTTGGGATATCTTGGAATCCCAATTTCAGGCTGGAAATAGCCCCAAAACAACCTGAGTTGGTCCAGAGAAGGGGCGCTGTGACCTACTGGGGACCCATGAAGGCCCCTGTCTTTCAGTAGCCTGTTTGCACAGCAGGTCCAAGGCTCAGCGAGGATTATGGGACCGGGAGGTGGAGACCCCAGCATGGCCTACAGTGGATAGAATCCATTTCCTCCATCCCCACCCCCAGGCCCCCAGGACCTGCATGCCCTCACACTGCCCACGTGTCCACTGGGTGGACACTCTGGGCCTGGCTCTGTCCTCAGAGTCTGCAGGCTCCCGCTCTTGCTCCCTGTGTCATCCTGGGAGTGCCCCCCTTCCCTTGAGAGGACCAGGCTGGGGTCCAGGTTTGCAGGAAGACCTCTGGGTGAAAGGCTCCCGCAGGTCAGAACCACTAGGGTGCACAGTGTACCCGCAAGCCCTTGGCTGGGGCTTAGGACCTGGAGGGCAGCTGGGAAGCAGGCATGTCCCTCTCCTTTCCCATTGTCCACTCACGCGTCTGTTGTCTTTGCACAGATGCTCTCTGCGTGCGACCAGGGAGGATGCGGGGGACAGGAGAGTTGCACTGAGCTGCTGAGGAGCAGGGTAGGAAGAGGCAGCACAGGACCACGGGGAGAGGAGGTGGACCCGAGAGCCTGCTGGTCGAATGGGAGCTACGGCTGCCGGCCTACACCATAGCCACAGAGACACCGGATCCTTAATCCACTGAGCCAGGCCAGGGATGGAAACCGCAACCTCATGGTTCCTGGTCGGATTGTTTCCGAAGCCCCACGATGGGAACTCCTGTTGGCTTTTCCTTTCAACCTCACCCAGACGGCCTTTCGGGAGCTGACGCGGCCACTTTCTTCCTCTAGCTGGCTCTGGATCTCACCGGACTCTGAGAGGGACCTCGGGATTTGGATCATCAACCAGTAGGGTGTCAGGGTCTTCGCTGTGCACACTTTCTGTCCTGCCTCCAGGGGTGTGCAGTGTGAACGAGGGTCAGGCCGGGCACAGAGTCAGGCCGCAGCTGCAGACCAGCACTCCAAGGCCCGGAACCGGAGGGGAAGCTGAGGCTCCCAAAGACGCAGGCAGCGCCCTCCTCAAGGCTGCAGGGAGCTGGGCTGCTGATCTCAGGGTGCGCCACCTTTGGCCTGGGATCCTACACCATCCAGTATCTCCGCAGGTCCTGAGCTGTCCTGCCCCGAACCCCGCGATGAAGCCGTTCCCCCTGTCCCCCTCCACCCGGGGCACAGCCTGGGTAGGGGCGTCTCTGCAGTCCTGGGCCCAGCGGGTGCTTCAGCAAACCCAGACATAGATCCAATAAGGGGACCAGGGCCCTGGGACTGGTGACCCGAGTTGCGCTGGACTGACCCGGACGACCCTCTCGCTCGCGGGCCGCGTCGCCCGGGTCTGCTCCTCCACCTCCGTTCCTGGGCCTCGGCTCCGCCCCACCGCCCGTCTCGCTGTGACCCCGCC

MSTRG.9843

ATATTTCAGAGTTATATTATTATATAAATAAAATGAAAATTTTGCAATATGAAAATTTTTCATATAGAATTATTTATTTTTCACTACCATTTATAAAGTTTTGCCTTTTAAGAGTTGGTAATGTTGGTAAATGTAGTAAAGTGTCAATAATTCAGCATTTAAAAGATATTTTGCTGTTGGAATATATGTGATTTCCTTTTGAGTCTGTGAGTAGTACACTAACACTTGCAGTAGCATTTTACAAATATTATTTTATGTTTGCAATAACCATATGAACTTAGTAGGGTATCTGCACTTTAAAACTTAGTAGGGTATCTGCACTTTAAAAGCAAGGAAATAAAATAAATAGACTCCAAACATTGATGGGACTGATTGCCCAAGACCACCCCAAAATAGATCCCAGCTAGAATGTAACCTCCTGAGGTAAAATTCAGAGTTCTTTCCACTATGCCACACTCCCTTCAAACAGTCTTTCATTCACTTGTTCAATAAATATCTCTTGAGTGCTTACTGTGTGCTATGTGTAGGTGCTGGGGAGAAAGTGGTCTCTAATTTAGAGACAGGGTGGGATAGGACATAGAAAGATTGTCATGGAAGTTCTCTGAGGAAGTGATACCTGGAAAATGTATAAGGAATAAACCAGGAAGGAAGTGAAAGAATTCAATCCAGGAAGAGGGAATAGGGTGGAGAATGAGTGAGCTTCAAGCAGGAAGAATTAAAAGGCCAGTGTGACTAAAGCATTATTTAAAAGAGGAAAGGTGGCATTAAACAGGGCTGTCAGTCCAGGCAAGAAGAAACCATATGTGCCAAGAAGGGGTTCTGAACATAGTGGGAGGCCAGTGAAGGGTGCTCAGGCTCTATGCGCCATGCTACAAATCAGAACACTTCCCAAGAGGGGAAACATTAGCATAAACTGTTCAGCTCACTTCAGTGCATTTCCATTCTCTCCCAAGATCTTGTCACATAAAGTCCTGGATGCATTAGTTCCTTTCCAATTCTTCAAATATGCAGAGATATTTGGAGTTCCCATTGTGGCACTGCAGAAGTGAATCTGGCTAGTATCCATGAGGATGCAGCTTCAATCCCTGCCCTTGCTTGAGTTGGGGATCCAGCATTGCCATGAGCTGTGGTGTAGGTTGCAGATGCAGCTTGGATCCTGCATTGCTGTGGTGTAAGCTGGCAACTCCAATTCTACCCCTAGTCTGGGAACTTCCATATGCCACGGGTGTGGGCTGAAAAAGCAAATATATATATGTGGTTTGGTTTGGCTTTTATCCAGTTTTGGTAGTTGTCTTCAGCTGGTAGTTGGTCTGATACAAGCTAATCCAAAGCAAATCTTTTCTCACTTCCTCTGAAGACTGCTGTCACACCTCAGAGATAGTGTGGGTTCCAGACTCAAATTTACTCACACTCCAGGAAGTCAGGGATCCTGGTCTGTGCACTACTTCTTTTCCAGGTCTATCCAGAAGACTGCTGTACCCTCTCTTTCTCCCTCCTTGACTCATCCCCAGGCTCACCAGTTCTTCAAAGTCCCTCCTCCAAGGCAATACGGTTTTTTGTTTTTTGTTTTTGCAGTCTCTAGGTTTTTCCATAAACTCGGCATGGGGCACTACGTCGACTGTCTCATCGGAGGCCACGGCGACTAGCGGTTCCTGAGGGCAGCCGCAAGCAGTGCGAGACCCTAGGCGAGGAAGGACAATAACTTGGAATTCGGACTCGATTTCCGGCGGGACAGCCTCGCGCTGGGCCGCTGAGGCCCAGGGGTGGGGGCTCGGAGCCCGGCCCACAGGGAGGAAGGCGCTGCCCGCTGGGCTGCCTCTGCGGCCGCCTCCTCCTCCGACGCCGTCGCCCCTCCCCACCCCGCC
